# The Role of Myeloid Differentiation Factor 2 in Stroke: Mechanisms and Therapeutic Potential

**DOI:** 10.3390/biom15070961

**Published:** 2025-07-04

**Authors:** Deyuan Zhu, Jihu Zhao, Qian Chen, Qiong Liu, Yibin Fang

**Affiliations:** Translational Research Institute of Brain and Brain-like Intelligence, Department of Neurovascular Disease, Shanghai Fourth People’s Hospital, School of Medicine, Tongji University, Shanghai 200434, China; zhudeyuan@tongji.edu.cn (D.Z.); zhaojihu@tongji.edu.cn (J.Z.); chenqian1989@tongji.edu.cn (Q.C.)

**Keywords:** ischemic stroke, myeloid differentiation factor 2, neuroinflammation, neuronal death, therapeutic target

## Abstract

Stroke represents a significant public health burden, ranking as a leading cause of death and disability globally. The prevalence of stroke increases with age, with ischemic stroke accounting for nearly 87% of cases globally. The pathophysiology of stroke is characterized by neuronal injury, neuroinflammation, and oxidative stress, which exacerbate brain damage and hinder recovery. Myeloid Differentiation Factor 2 (MD2), an accessory protein of Toll-like receptor 4 (TLR4), has emerged as a key player in mediating inflammatory responses in stroke. This short review discusses the molecular mechanisms by which MD2 contributes to neuroinflammation and neuronal death following stroke and highlights MD2 as a promising therapeutic target for stroke treatment. Subsequently, we investigate the potential of MD2 inhibitors, their underlying mechanisms, and the therapeutic prospects of such inhibitors in reducing stroke-induced brain damage.

## 1. Introduction

Stroke represents a significant global health challenge. It is the second leading cause of mortality and a major contributor to the prevalence of long-term disability, particularly in older populations [1]. The two principal categories of stroke are ischemic and hemorrhagic, and they have distinct mechanisms but share common outcomes: significant brain damage [2,3], neuroinflammation [4], and the death of neurons and glial cells [5,6]. The predominant form, ischemic stroke, occurs due to the blockage of cerebral arteries, leading to oxygen and glucose deprivation in brain tissue [7]. The ischemic cascade, a sequence of events that follows ischemia, encompasses a number of processes, including the failure of cellular energy production, excitotoxicity, oxidative stress, and inflammation [8].

The role of neuroinflammation in stroke pathophysiology is now recognized as being both beneficial and detrimental [9]. While the inflammatory response is essential for clearing dead tissue and promoting repair [10], excessive or prolonged inflammation exacerbates neuronal injury and impairs recovery [11]. Toll-like receptor 4 (TLR4) has emerged as one of the central players in driving post-stroke inflammation [12,13]. TLR4, a member of the Toll-like receptor family [14], is essential in recognizing pathogen-associated molecular patterns (PAMPs) [15] and damage-associated molecular patterns (DAMPs) [16].

Myeloid Differentiation Factor 2 (MD2), the coreceptor for TLR4 [17], binds with these ligands to activate downstream inflammatory pathways [18,19]. MD2 is a glycoprotein weighing approximately 25–30 kDa that plays an indispensable role in TLR4 signaling [20]. Structurally, MD2 consists of a β-cup fold [18], which provides a hydrophobic pocket [21] that binds to the lipid A component of bacterial lipopolysaccharide (LPS) [22] as well as endogenous ligands released during tissue damage [23]. The TLR4-MD2 complex is capable of recognizing a diverse range of ligands, including PAMPs such as lipopolysaccharide (LPS) and DAMPs such as high-mobility group protein 1 (HMGB1), heat shock proteins, and oxidized phospholipids [24,25]. These endogenous ligands are released from injured or dying cells in the brain during ischemic stroke [23]. This short review aims to explore the role of MD2 in stroke pathophysiology, its contribution to neuroinflammation and neuronal death, and the potential therapeutic strategies targeting MD2 to mitigate stroke-induced brain damage.

## 2. MD2 Expression and Distribution in the Nervous System

**Figure 1:**
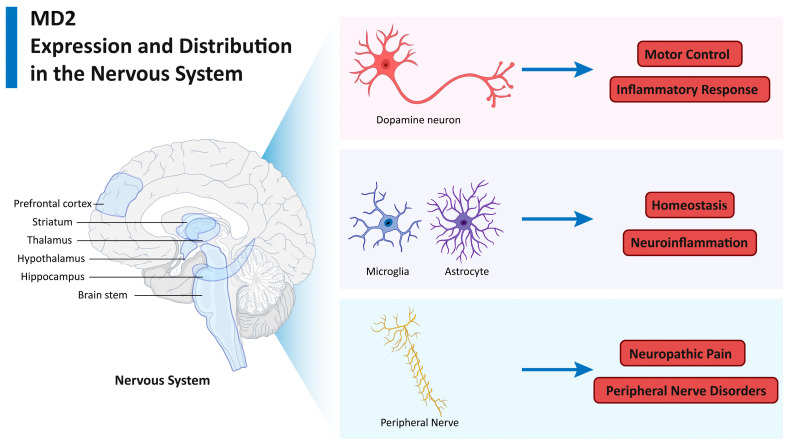
Expression and distribution of MD2 in the nervous system. This figure illustrates the expression and distribution of MD2 in various cell types within the nervous system. MD2 is expressed in neurons, including those in the prefrontal cortex, striatum, thalamus, hypothalamus, hippocampus, brain stem, etc. Following stress or injury, such as ischemic stroke or traumatic brain injury, neuronal MD2 expression increases, indicating its role in neuroinflammatory responses. Glial cells, including microglia, astrocytes, and oligodendrocytes, also express MD2, particularly under inflammatory conditions. Microglia exhibit high levels of MD2 during activation, facilitating DAMP recognition and TLR4 signaling. Additionally, MD2 is present in peripheral nerve cells, such as dorsal root ganglion neurons, suggesting its involvement in pain modulation and peripheral neuroinflammation. The comprehensive distribution of MD2 underscores its significant role in both central nervous system and peripheral nervous system inflammation and homeostasis.

MD2 is expressed at significant levels in numerous tissues throughout the body; however, its distribution within the nervous system has become an increasingly pertinent area of research interest, given its role in modulating neuroinflammatory responses [26,27]. A number of studies have demonstrated that MD2 is not confined to immune cells but is also expressed in a range of cell types within the nervous system (Figure 1) [28,29,30,31].

### 2.1. MD2 in Neurons

Initially, MD2 was thought to be predominantly expressed in microglia and astrocytes, the brain’s resident immune cells [28,30]. However, recent studies have demonstrated that neurons in various regions of the brain also express MD2, including the olfactory bulb, cortex, thalamus, red nucleus, brainstem reticular formation, and others [32,33]. Furthermore, MD2 expression is significantly elevated in dopaminergic neurons within the substantia nigra and ventral tegmental area, which extend projections to the striatum. This observation lends support to the notion that MD2 may play a role in motor control processes [33]. Following injury or stress, including during ischemic stroke and traumatic brain injury, neurons display an increased expression of MD2 [32]. This suggests that MD2 may be involved in the inflammatory response of neurons.

### 2.2. MD2 in Glial Cells

MD2 is markedly expressed in microglia, where it mediates responses to DAMPs and promotes the release of pro-inflammatory cytokines [30,34]. Following stroke or central nervous system (CNS) injury, activated microglia express high levels of MD2, which facilitates the recognition of DAMPs, leading to the activation of TLR4 signaling [35,36]. In addition to microglia, astrocytes [28] and oligodendrocytes [29] have been shown to express MD2 under inflammatory conditions. The presence of MD2 in these glial cells suggests a broader role in CNS homeostasis and inflammation beyond the traditional microglial context.

### 2.3. MD2 in Peripheral Nerve Cells

In addition to its expression in the CNS, MD2 has been detected in peripheral nerve cells, including the dorsal root ganglion (DRG) neurons [37]. This peripheral expression highlights the potential role of MD2 in pain modulation and peripheral neuroinflammatory conditions [37,38]. Its presence in the DRG suggests that MD2 might contribute to neuropathic pain and other peripheral nerve disorders by amplifying inflammatory responses.

## 3. TLR4-MD2 Complex and Stroke

**Figure 2:**
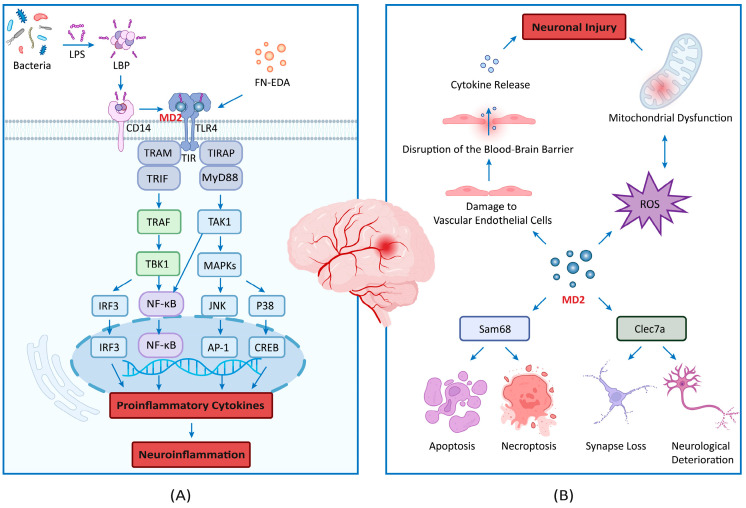
The role of the TLR4-MD2 complex and TLR4-independent mechanisms in stroke. This figure illustrates the dual contributions of MD2 in stroke pathophysiology. (**A**) highlights the TLR4-MD2 complex’s role in neuroinflammation and neuronal death, where MD2 facilitates TLR4 dimerization and activates MyD88- and TRIF-dependent pathways, leading to NF-κB and IRF3 activation. It is worth noting that the MyD88 pathway activates TAK1, which in turn drives the activation of NF-κB. Furthermore, TAK1 promotes the activation of the MAPK cascade (JNK/p38). Activated MAPKs then phosphorylate AP-1, while TRIF-dependent signaling activates IRF3. Collectively, these transcription factors induce the release of pro-inflammatory cytokines, blood–brain barrier disruption, and neuronal apoptosis/necroptosis. (**B**) explores TLR4-independent roles of MD2, including its interactions with Sam68, promoting apoptosis and necroptosis, and with Clec7a, which exacerbates synaptic loss through microglial engulfment. MD2 also contributes to reactive oxygen species production and blood–brain barrier permeability, highlighting its broader role in stroke-induced damage and its potential as a therapeutic target.

The TLR4-MD2 complex plays a significant role in stroke by contributing to neuroinflammation and ultimately leading to neuronal cell death. The activation of this complex can trigger pro-inflammatory signaling cascades that exacerbate the inflammatory response post-stroke, potentially worsening the outcome for affected neurons. Understanding the involvement of the TLR4-MD2 complex in neuroinflammation and neuronal cell death post-stroke may provide insights into novel therapeutic strategies for mitigating the detrimental effects of stroke on the brain (Figure 2A).

### 3.1. Molecular Mechanisms

Following ligand binding, MD2 plays a role in the dimerization of TLR4 receptors, which in turn leads to the formation of the TLR4-MD2 signaling complex [18,21]. This dimerization is crucial for initiating downstream signaling cascades [18]. MD2 plays an indispensable role in the stabilization of the TLR4-LPS or TLR4-DAMP complex, which is vital for the transmission of pro-inflammatory signals that ultimately lead to the production of cytokines such as tumor necrosis factor-alpha (TNF-α), interleukin-6 (IL-6), and interleukin-1 beta (IL-1β) [20].

The TLR4-MD2 complex has the capacity to activate two discrete signaling pathways, namely the MyD88-dependent pathway [39] and the MyD88-independent (Toll–interleukin receptor homology-domain-containing adapter-inducing interferon-β (TRIF)-dependent) pathway [40]. The MyD88 pathway recruits TAK1, which drives the activation of both NF-κB and the MAPK cascade. Activated MAPKs then phosphorylate AP-1, while NF-κB transcribes pro-inflammatory cytokines. Meanwhile, the TRIF pathway activates both IRF3 and NF-κB, thereby inducing type I interferons and pro-inflammatory mediators. Thus, both pathways converge on NF-κB activation while differentially engaging MAPK (MyD88) and IRF3 (TRIF) effectors to amplify neuroinflammation [40,41,42].

Research has found that the extra domain A of fibronectin (FN-EDA) activates the TLR4-MD2 complex by directly binding to the central and C-terminal regions of TLR4 [43]. This interaction results in the activation of inflammatory signaling pathways, which in turn contribute to the progression of a number of different diseases, including atherosclerosis [44], diabetes [45], ischemic stroke [46], and myocardial reperfusion injury [47]. Molecular docking experiments revealed that the sequence “SPEDGIRELF” in FN-EDA is the core of its interaction with TLR4 [43]. Molecular dynamics simulations further confirmed the stability of the FN-EDA-TLR4-MD2 complex [43]. FN-EDA has been demonstrated to bind to the variable region of TLR4, thereby eliciting robust pro-inflammatory signaling.

### 3.2. Neuroinflammation

Neuroinflammation represents a complex and pivotal process in the pathophysiology of stroke, impacting both the acute injury and chronic neurological deficits associated with this condition [48]. The activation of immune cells, such as microglia and infiltrating macrophages [49], is a key component of neuroinflammation in stroke [50]. These immune cells respond to signals released by dying neurons and glial cells, detecting DAMPs via TLR4 and its co-receptor MD2 [34].

Following ischemic stroke, there is the immediate and substantial activation of microglia and macrophages, both of which play a role in triggering and maintaining neuroinflammation [4]. These immune cells play a critical role in the recognition of danger signals and the subsequent inflammatory response [26]. The presence of MD2 is essential for the activation of TLR4 and the detection of DAMPs, underscoring the importance of this complex in the pathogenesis of stroke-induced neuroinflammation [51,52].

During the acute phase of stroke, neuroinflammation is predominantly detrimental to the brain [4]. The activation of microglia and macrophages results in the release of a multitude of pro-inflammatory cytokines, chemokines, and ROS, which serve to exacerbate tissue damage and disrupt the blood–brain barrier [52,53]. Microglia and astrocytes exist on a continuum of activation states, influenced by cytokines like TGF-β and IL-10. These states modulate neuroinflammation through TLR4-independent pathways, such as TNF-α/IL-6 synergy, which exacerbates neuronal damage [4,9]. This inflammatory cascade is orchestrated by various signaling pathways, including the NF-κB pathway [54] and the NOD-like receptor protein 3 (NLRP3) inflammasome [55], leading to the generation of inflammatory mediators that sustain the neuroinflammatory response.

### 3.3. Neuronal Death

The role of MD2 in neuronal death is multifactorial. First, the activation of the TLR4-MD2 complex in neurons leads to the upregulation of pro-apoptotic genes through the activation of NF-κB and other transcription factors [56]. The upregulation of TLR4-MD2 activation enhances apoptosis through pro-apoptotic genes such as Bax, Bak, Bid, and Fas/FasL, mediated by transcription factors including NF-κB, AP-1, STAT1/3, IRF3/7, and p53. Recent studies further highlight the NLRP3 inflammasome and cGAS-STING signaling in amplifying TLR4-driven apoptosis. These mechanisms are pivotal in both inflammatory and oncogenic contexts [56,57].

TLR4-MD2 signaling activates both apoptotic and pyroptotic pathways. In apoptosis, caspases-3 and -9 are triggered via mitochondrial cytochrome c release [56]. In pyroptosis, cytosolic LPS detection activates the non-canonical inflammasome pathway, engaging caspases-4/5/11 (human/murine homologs), which cleave gasdermin D to drive pore formation and IL-1β/IL-18 maturation [57]. Caspase-8 is indirectly activated through TRIF-RIPK1 complexes, amplifying inflammatory signaling [58]. These pathways collectively contribute to neuronal death and systemic inflammation in stroke models [57,58].

In addition to its role in apoptosis, MD2 has also been demonstrated to contribute to necroptosis, a form of programmed necrosis that is mediated by the receptor-interacting protein kinases (RIPK1 and RIPK3) [59]. MD2 promotes necroptosis by enhancing TLR4-mediated signaling. Upon LPS stimulation, MD2/TLR4 activates downstream pathways that induce RIPK1 phosphorylation. When apoptosis is blocked, phosphorylated RIPK1 interacts with RIPK3 via RHIM domains, forming a necrosome that phosphorylates MLKL, leading to plasma membrane disruption and DAMP release [59,60].

MD2 also contributes to neuronal death via the production of reactive oxygen species (ROS) [61]. The activation of TLR4-MD2 signaling leads to the generation of ROS through the action of nicotinamide adenine dinucleotide phosphate (NADPH) oxidases and mitochondria [62,63]. TLR4-MD2 signaling activates NADPH oxidases (NOX2/4) via PKC-δ, while mitochondrial dysfunction arises from impaired electron transport chain activity, collectively increasing ROS [61,62]. Excessive ROS production causes oxidative damage to lipids, proteins, and DNA, leading to neuronal injury and death [53,64]. The interaction between oxidative stress and inflammation results in the creation of a toxic environment within the ischemic brain, which serves to further promote cell death.

## 4. TLR4-Independent Mechanisms of MD2 in Stroke

While MD2’s primary role is in the activation of the TLR4 signaling pathway, growing evidence suggests that MD2 also influences TLR4-independent mechanisms that contribute to neuroinflammation and neuronal injury [32,65]. These TLR4-independent mechanisms represent an emerging area of interest in neuroprotection, especially in conditions like ischemic stroke and traumatic brain injury, where TLR4 activation might not be the sole contributor to damage. This suggests that MD2 plays a broader role in neuroinflammation and neuronal death beyond its interaction with TLR4, making it an even more attractive target for therapeutic intervention. (Figure 2B)

### 4.1. MD2 and Sam68 Interaction

Under conditions of neuronal injury, particularly excitotoxicity triggered by the excessive activation of glutamate receptors (e.g., NMDA receptors), MD2 expression is significantly upregulated, and critically, its subsequent pro-death effects occur independently of TLR4. The upregulated MD2 directly interacts with the nuclear RNA-binding protein Sam68, which is known to regulate apoptosis. This interaction serves as a critical trigger, inducing the translocation of Sam68 from the nucleus to the cytoplasm. Within the cytoplasm, translocated Sam68 functions as an essential signaling adaptor, bridging MD2 to downstream death effectors. This engagement simultaneously activates two distinct programmed cell death pathways. Apoptosis is mediated through the cleavage of caspase-8 (extrinsic apoptosis) and caspase-3 (intrinsic apoptosis), while necroptosis is driven by the phosphorylation of RIPK3 and MLKL [32,66]. Thus, the MD2-Sam68 interaction, with the pivotal nuclear-to-cytoplasmic shift in Sam68, is central to inducing neuronal death during NMDA receptor-mediated excitotoxicity. Substantial evidence confirms this pathway’s role, and importantly, the targeted disruption of the MD2-Sam68 interaction confers significant neuroprotection against ischemic neuronal injury, highlighting its potential as a therapeutic target [67,68].

### 4.2. MD2 and Clec7a Interaction

Emerging research suggests that Clec7a, a C-type lectin receptor, may interact with MD2-related signaling pathways [65]. While Clec7a is primarily involved in fungal recognition [69], it was found to have an impact on long-term neurological damage after ischemic stroke, specifically manifested through exacerbating synapse engulfment mediated by microglia, leading to synapse loss and neurological deterioration [65,70]. Furthermore, one study also revealed an interaction between Clec7a and MD2 in neurons, and it seemed that MD2 may be the ligand of microglial Clec7a.

The interaction between Clec7a and MD2 drives microglia-mediated synaptic phagocytosis, exacerbating post-stroke neurological deficits. Clec7a recognizes MD2 as a ligand, triggering the microglial engulfment of excitatory synapses via phagocytosis pathways. This interaction promotes synaptic loss, neuronal hyperexcitability, and cognitive impairment. The genetic or pharmacological inhibition of Clec7a reduces MD2-dependent synaptic elimination, preserves synaptic density, and improves long-term functional recovery. The Clec7a-MD2 axis thus represents a critical pathway in post-stroke synaptic pathology, highlighting its therapeutic potential for mitigating neurodegeneration and neurological dysfunction [70]. This discovery indicates the crucial role of Clec7a in ischemic stroke and suggests it may be a potential target for treating ischemic stroke.

### 4.3. MD2 and ROS

MD2 has been implicated in the production of ROS, a major contributor to neuronal damage during oxidative stress [71]. ROS production following MD2 activation has been observed in models of ischemic stroke, where oxidative damage exacerbates neuronal injury [53,64]. While TLR4 signaling is often involved in this process, recent studies suggest that MD2 can modulate ROS production independently of TLR4, particularly in the presence of mitochondrial dysfunction [53,64]. Under cellular stress, MD2 translocates to mitochondria, binding mitochondrial DAMPs such as cardiolipin and mitochondrial DNA. This interaction destabilizes the inner mitochondrial membrane, increasing permeability transition pore opening and disrupting electron transport chain complexes I and III, leading to electron leakage and superoxide generation [64,71]. Concurrently, MD2 recruits and activates NOX2/NADPH oxidase at mitochondrial membranes, amplifying ROS production [62,71]. Furthermore, MD2-TLR4 dissociation enables MD2 to activate redox-sensitive kinases (e.g., JNK/p38 MAPK), perpetuating ROS via NF-κB and NLRP3 inflammasome pathways [56,57]. These TLR4-independent mechanisms collectively exacerbate oxidative damage in post-stroke neurodegeneration.

### 4.4. MD2 and Blood–Brain Barrier Disruption

One critical aspect of neuroinflammation is the disruption of the blood–brain barrier (BBB), which allows peripheral immune cells to infiltrate the CNS [72]. MD2 has been shown to play a role in BBB disruption independently of TLR4 by interacting with vascular endothelial cells and promoting cytokine release [26,73]. This results in the increased permeability of the BBB, thereby facilitating the entry of immune cells into the brain and the exacerbation of inflammation [74,75]. MD2 contributes to BBB disruption by interacting with vascular endothelial cells and promoting the release of cytokines such as IL-6 and TNF-α. This process involves the activation of NF-κB and upregulation of matrix metalloproteinases (e.g., MMP-9), which degrade tight junction proteins (e.g., claudin-5, occludin) [72,73]. Additionally, MD2 may facilitate ROS-mediated endothelial dysfunction, further compromising BBB integrity [64]. The exact signaling pathways involved in this process remain under investigation, but they represent an important area for therapeutic development.

## 5. Therapeutic Targeting of MD2 in Stroke

Given its central role in neuroinflammation and neuronal death, MD2 is a promising therapeutic target for stroke. Several strategies have been developed to inhibit MD2 function, including small-molecule inhibitors, peptides, and biologics. These inhibitors aim to disrupt the interaction between MD2 and TLR4 or inhibit MD2 function, thereby reducing the activation of downstream inflammatory pathways and protecting against brain damage [32,76,77,78,79] (Table 1).

### 5.1. Small-Molecule Inhibitors

Research indicates that AM1241 (a selective cannabinoid receptor 2 agonist [81]) has the capacity to markedly suppress the generation of pro-inflammatory cytokines (such as TNF-α and IL-6) and oxidative stress [82]. AM1241 directly binds to MD2, inhibiting the formation of the TLR4-MD2 complex [76]. The activation of this complex is crucial for promoting inflammation and oxidative stress responses [8]. Through molecular docking experiments and surface plasmon resonance experiments, the direct interaction between AM1241 and MD2 was confirmed [76]. The formation of the TLR4-MD2 complex activates the MAPK and NF-κB signaling pathways, leading to inflammation and cell apoptosis [83]. AM1241 inhibits these signaling pathways, thereby alleviating the inflammatory response and neuronal apoptosis [76]. In a mouse model of cerebral ischemia–reperfusion injury (CIRI), AM1241 significantly improved neurological function damage by reducing brain edema, infarct volume, and cell apoptosis, demonstrating its powerful neuroprotective effect [76].

Nalmefene, an opioid derivative, has also been shown to inhibit TLR4 signaling by binding to MD2 [77]. Nalmefene reduces the inflammatory response in models of ischemia–reperfusion injury and prevents neuroinflammation by blocking the interaction between TLR4 and MD2 [77]. Interestingly, Nalmefene does not exhibit enantioselectivity, meaning that both its (+) and (−) isomers have similar effects on TLR4-MD2 inhibition [77].

### 5.2. Peptide Inhibitor

Peptide inhibitors that target MD2 have emerged as a promising alternative to small-molecule inhibitors. These peptides are designed to mimic the binding interface between MD2 and TLR4, competitively inhibiting the formation of the TLR4-MD2 complex. One such peptide, Tat–cold-inducible RNA binding protein (Tat-CIRP), has shown neuroprotective properties in animal models of stroke [32,84]. Tat-CIRP inhibits MD2 function, reducing the activation of NF-κB and other pro-inflammatory pathways, thereby mitigating neuroinflammation and neuronal death [32,79].

Peptides offer several advantages over small-molecule inhibitors, including greater specificity and the ability to cross the BBB [79]. However, challenges remain in optimizing peptide stability and bioavailability. Peptides are often rapidly degraded by proteases in the bloodstream, limiting their therapeutic efficacy [85]. Strategies to improve peptide stability, such as the use of modified amino acids or peptide conjugation, are currently being explored.

### 5.3. Natural Compound

Natural compounds that target MD2 have also garnered attention for their potential neuroprotective effects. Stepharine, an alkaloid derived from *Stephania japonica* (Thunb.) Miers [86], has been found to possess a potent inhibitory effect on excessive microglial activation [87]. Studies have shown that Stepharine has the potential to mitigate neurological impairment and minimize brain injury in rats with middle cerebral artery occlusion (MCAO) [80]. Additionally, Stepharine has been observed to prevent neuronal loss and suppress exaggerated microglial activation [80]. This effect may be attributed to its direct interaction with the TLR4-MD2 complex, which results in reduced TLR4 expression and the attenuation of NF-κB signaling pathway activation. Additionally, there is the inhibition of pro-inflammatory mediator expression [80]. These findings suggest that Stepharine may represent a promising natural compound for the treatment of ischemic conditions and the improvement of stroke recovery outcomes. Natural compounds like curcumin and resveratrol also exhibit MD2-modulating properties. Curcumin binds MD2 to inhibit NF-κB activation [82], while resveratrol suppresses TLR4/MD2 signaling and oxidative stress [8]. However, these studies remain limited, and the mechanistic links to MD2 require further validation to establish causality.

## 6. Conclusions and Future Directions

MD2 is a pivotal regulator of neuroinflammation and neuronal death in ischemic stroke, acting through both TLR4-dependent and independent pathways. While MD2 inhibition offers therapeutic promise, its complex interactome necessitates innovative strategies like nanocarriers for timely BBB penetration during the critical 6–12 h post-stroke window. Future efforts should focus on the following: elucidating MD2’s context-specific signaling crosstalk, designing rapid CNS delivery systems, and combining MD2 inhibitors with neuroprotective agents to achieve precision stroke therapy.

## Figures and Tables

**Table 1:** Therapeutic targeting of MD2 in stroke.

Inhibitors	Type	Function	Effects	Models	Reference
Tat-CIRP	Peptide	Competitively binds to MD2	Disrupts MD2-TLR4 binding, reduces neuroinflammation and neuronal death	MCAO mice, brain hemorrhage mice, I/R injury rhesus monkey	[32]
AM1241	Small Molecule	Directly binds to MD2	Inhibits the formation of the TLR4-MD2 complex, alleviates the inflammatory response and neuronal apoptosis	CIRI mice	[76]
Nalmefene	Small Molecule	Binds to MD2	Prevents neuroinflammation and brain damage by blocking the interaction between TLR4 and MD2	Microglia BV-2 cell line	[77]
Stepharine	Natural Compound	Binds to TLR4-MD2 complex	Improved outcomes in MCAO rats, reduces neuronal loss, suppresses microglial overactivation via the inhibition of the TLR4/NF-κB pathway	MCAO rats	[80]

MD2: Myeloid Differentiation Factor 2; TLR4: Toll-like receptor 4; MCAO: middle cerebral artery occlusion; I/R: ischemia/reperfusion; CIRI: cerebral ischemia–reperfusion injury.

## Data Availability

No new data were created.

## Abbreviations

The following abbreviations are used in this manuscript:

MD2	Myeloid Differentiation Factor 2
TLR4	Toll-like receptor 4
DAMPs	Damage-associated molecular patterns
PAMPs	Pathogen-associated molecular patterns
NF-κB	Nuclear factor-kappa B
ROS	Reactive oxygen species
CNS	Central nervous system
BBB	Blood–brain barrier
TNF-α	Tumor necrosis factor-alpha
IL-1β	Interleukin-1 beta
IL-6	Interleukin-6
LPS	Lipopolysaccharide
TRIF	TIR-domain-containing adapter-inducing interferon-β
IRF	Interferon regulatory factor
MyD88	Myeloid differentiation primary response 88
NADPH	Nicotinamide adenine dinucleotide phosphate
RIPK	Receptor-interacting protein kinase
HMGB1	High-mobility group box 1
NLRP3	NOD-like receptor family pyrin domain-containing 3
RIPK1/RIPK3	Receptor-interacting serine/threonine–protein kinase 1/3
DRG	Dorsal root ganglion
NMDA	N-methyl-d-aspartate
FN-EDA	Extra domain A of fibronectin
Tat-CIRP	Tat–cold-inducible RNA binding protein
MCAO	Middle cerebral artery occlusion
CIRI	Cerebral ischemia–reperfusion injury
